# HIV-Prevalence in Tuberculosis Patients in Germany, 2002–2009: An Estimation Based on HIV and Tuberculosis Surveillance Data

**DOI:** 10.1371/journal.pone.0049111

**Published:** 2012-11-07

**Authors:** Lena Fiebig, Christian Kollan, Barbara Hauer, Barbara Gunsenheimer-Bartmeyer, Matthias an der Heiden, Osamah Hamouda, Walter Haas

**Affiliations:** 1 Respiratory Infections Unit, Department for Infectious Disease Epidemiology, Robert Koch Institute, Berlin, Germany; 2 HIV/AIDS, STI and Blood-borne Infections Unit, Department for Infectious Disease Epidemiology, Robert Koch Institute, Berlin, Germany; McGill University, Canada

## Abstract

Tuberculosis (TB) and HIV comorbidity is a major challenge in TB prevention and control but difficult to assess in Germany as in other countries, where data confidentiality precludes notifying the HIV status of TB patients. We aimed to estimate the HIV-prevalence in TB patients in Germany, 2002–2009, and to characterize the HIV/TB patients demographically. Data from the long-term observational open multicentre cohort ClinSurv HIV were used to identify incident TB in HIV-positive individuals. We assessed the cohort’s coverage for the nationwide HIV-positive population by contrasting ClinSurv HIV patients under antiretroviral therapy (ART) with national HIV patient numbers derived from ART prescriptions (data by Insight Health; available for 2006–2009). The HIV-prevalence in TB patients was calculated as the number of HIV/TB cases projected for Germany over all culture-positive TB notifications. From 2002 to 2009, 298 of 15,531 HIV-positive patients enrolled in the ClinSurv HIV cohort were diagnosed with TB. A 21% cohort coverage was determined. The annual estimates of the HIV-prevalence in TB patients were on average 4.5% and ranged from 3.5% (95%CI 2.3–5.1%) in 2007 to 6.6% (95%CI 5.0–8.5%) in 2005. The most recent estimate for 2009 was 4.0% (95%CI 2.6–5.9%). The 298 HIV/TB patients were characterized by a male-to-female ratio of 2.1, by a median age of 38 years at TB diagnosis, and by 59% of the patients having a foreign origin, mainly from Subsahara Africa. We provide, to our knowledge, the first estimate of the HIV-prevalence in TB patients for Germany by joint evaluation of anonymous HIV and TB surveillance data sources. The identified level of HIV in TB patients approximates available surveillance data from neighbouring countries and indicates a non-negligible HIV/TB burden in Germany. Our estimation approach is valuable for epidemiological monitoring of HIV/TB within the current legal frameworks.

## Introduction

Tuberculosis (TB) and HIV comorbidity is a relevant public health issue worldwide and does not spare the European region [1]–[3]. HIV/TB is considered a leading cause, together with multi- and extensively drug resistant tuberculosis (M/XDR-TB), impairing success in TB control [4], [5]. Both HIV and TB adversely interact when affecting the same patient: TB as an AIDS-defining disease is a major cause of morbidity and mortality in people living with HIV (PLWH) [1], [6]. HIV-positive individuals infected with *Mycobacterium (M.) tuberculosis* complex bacteria are placed at a 20 to 30 times greater risk of developing active TB [7]–[9]; moreover, they are more prone to endogenous reactivation or exogenous reinfection than HIV-negative individuals [7], [8], [10], [11]. Diagnosis of latent infection and active TB is more challenging in immunocompromised individuals including PLWH [12]. They may present with atypical clinical manifestations of TB or have masked disease with implications on adequate clinical case management [12], [13].

The need for systematic HIV/TB surveillance including the monitoring of the level of routine testing has been internationally recognized. The strategic environment was decisively shaped by the “Interim policy on collaborative TB/HIV activities” launched in 2004 by the WHO [14] (latest update in 2012 [15]) advocating three main areas of activities: A. establishing the mechanisms for collaboration between TB and HIV programmes; B. decrease the burden of TB in PLWH; and C. decrease the burden of HIV in TB patients.

The European region was specifically addressed with the “Berlin Declaration on Tuberculosis” of the WHO European Ministerial Forum [5], the “European Framework to decrease the burden of TB/HIV” [16], and the “Framework Action Plan to fight tuberculosis in the European Union” [17], adding authority on collaboration between TB and HIV/AIDS national programmes and joint surveillance. Through the “Global Plan to Stop TB” all WHO member states were requested to provide information on HIV-prevalence among new TB notifications by the year 2010 [18]. However, levels of implementing routine HIV/TB surveillance have remained heterogeneous in European Union/European Economic Area (EU/EEA) member states. For 2010, only 18/30 EU/EEA member states, excluding Germany, provided data on HIV among TB patients to the European Centre for Disease Prevention and Control (ECDC) [19].

In Germany, both HIV infections and active TB are of recognized public health relevance [20]. Mandatory notification according to the German Protection against Infection Act [21] resulted in 3.5 new HIV cases and 5.3 active TB cases per 100,000 population in 2011 [22]. However, their reporting channels are separate: TB cases are notified by name to local public health offices and anonymously transmitted via State health departments to the national level, the Robert Koch Institute (RKI). HIV diagnoses are anonymously notifiable directly to the RKI. Data confidentiality legislation precludes recording the HIV status of TB patients in national TB notification data. HIV surveillance instruments may however include information on TB as AIDS-defining disease.

With this study, we aimed to estimate the HIV-prevalence in TB patients in Germany from 2002 to 2009 by jointly evaluating available anonymous HIV and TB surveillance data sources, as well as to characterize HIV/TB patients demographically.

## Materials and Methods

The rationale of our estimation of the HIV-prevalence in TB patients was to use an HIV surveillance instrument to identify HIV/TB case numbers and relate them to TB notification data as subsequently described.

### HIV/TB Case Definition

We defined eligible HIV/TB patients as HIV-diagnosed individuals living in Germany, irrespective of antiretroviral treatment (ART) status, who presented with active culture-positive TB (all forms) from 2002 to 2009. Individuals with history of previous TB only were not eligible.

### Data Sources

**Figure 1:**
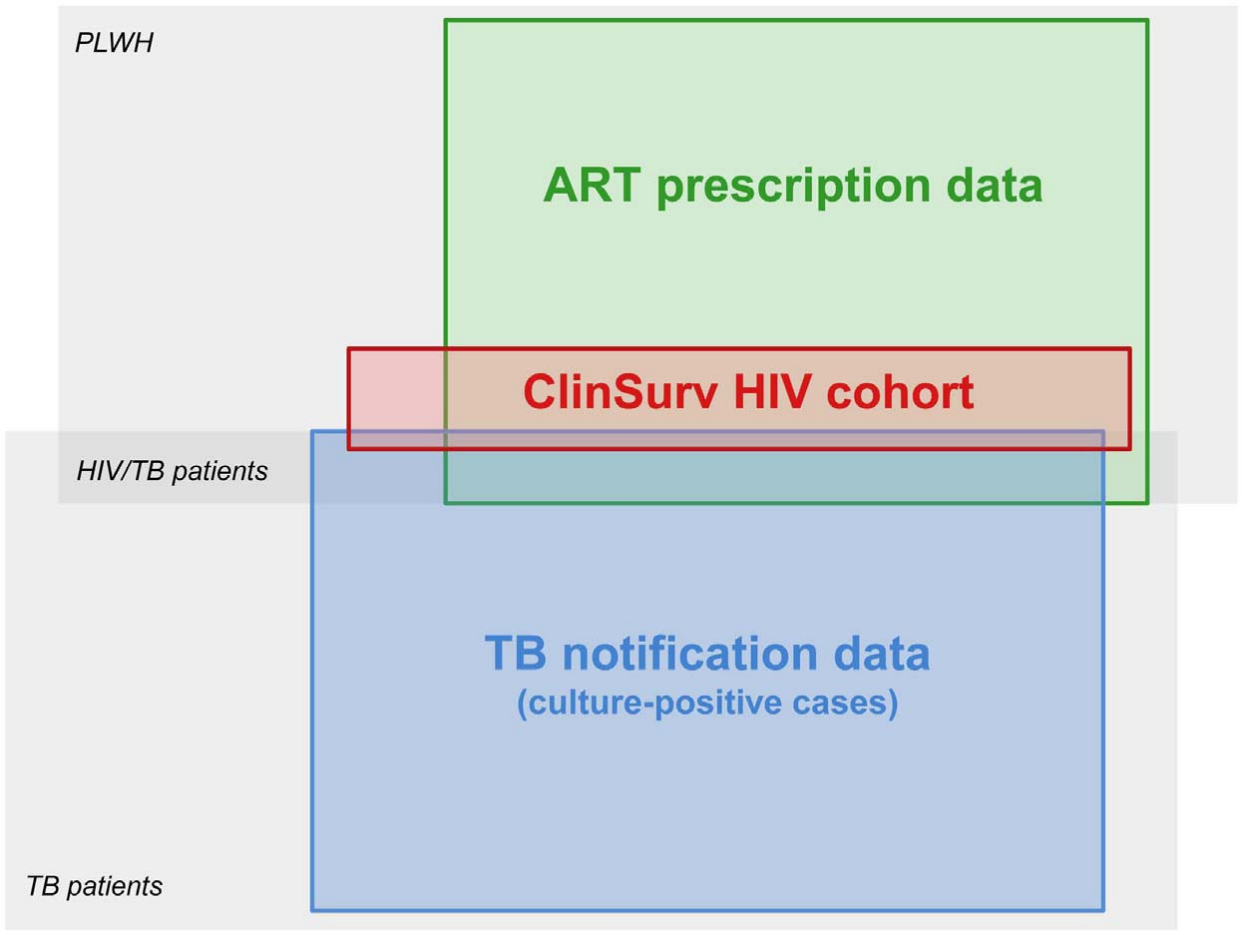
Schematic overview on subpopulations and available data sources in Germany. Subpopulations of interest (*in italic*) include PLWH, mostly placed under ART after HIV diagnosis, as well as TB and HIV/TB patients. Data sources (coloured boxes) include national TB notification data restricted to culture-positive cases, ART prescription data by Insight Health, and the ClinSurv HIV cohort. TB notification and ART prescription data capture patients irrespective of their comorbidity status. ClinSurv HIV is the only source systematically capturing both HIV and TB. This schematic is not to scale.

The investigated data sources comprised (i) the ClinSurv HIV cohort; (ii) ART prescription data; and (iii) national TB notification data of Germany (Figure 1):

- The ClinSurv HIV cohort is a prospective open multicentre observational cohort study for the clinical surveillance of HIV [23], [24]. The cohort has been established in 1999 and is maintained by collaborating HIV clinical centres in Germany, mainly located in urban areas, and the RKI as coordinating centre. All HIV-positive individuals presenting in one of these clinical centres are by default enrolled in the cohort. The data set comprises anonymous demographic patient information (age, sex, geographic origin) and time-related variables on clinical events, such as diagnoses of AIDS-defining diseases including TB [25], as well as the administered ART regimen. TB is defined by a positive culture of *M. tuberculosis* complex and includes both pulmonary and extra-pulmonary forms. The patients’ origin was assessed as foreign, when their predominant place of residence had been abroad (supported by information on a foreign country of birth or foreign citizenship), and was classified as in the European HIV Infection Data Set (EHIDS) [26].
- ART prescription data comprise prescribed daily drug doses recorded by quarter and by Federal State of Germany. The data set was made available by the service provider Insight Health for 2006 to 2009.
- National TB notification data result from mandatory notification of active TB (all forms) with indication of antituberculotic therapy, irrespective of laboratory confirmation [27]. For agreement with the ClinSurv HIV cohort, the TB notification data set was restricted to culture-positive cases.

All data sources were anonymous and independent from each other; case-based record-linkage was not performed.

### Estimation Approach

Our estimation consisted of three major steps: step 1 was to extract TB cases among patients enrolled in ClinSurv HIV; step 2 was to assess the nationwide coverage of the ClinSurv HIV cohort; and step 3 to determine the HIV-prevalence in TB patients in Germany based on results from steps 1 and 2.

In step 1, the ClinSurv HIV cohort database was searched for all patients newly diagnosed with TB from 2002 to 2009. These annual HIV/TB case numbers were provided with a 95% binomial exact confidence interval (CI) using the number of all HIV-diagnosed patients presenting at the clinical centres in the respective years as a base.

To assess the nationwide coverage of the ClinSurv HIV cohort in step 2, we focussed on patients under ART. To quantify this subpopulation, ART prescription data, a data source independent from HIV notification data and thus not subject to physicians’ reporting behaviour [28], were used. The approach to derive patient numbers from ART prescription data was established by Kollan et al. [29]. An underlying assumption was that ART regimen and treatment interruptions as recorded in ClinSurv HIV apply likewise to patients outside of the cohort. To account for patients without statutory health insurance (private, uninsured and free medical care), whose prescriptions are not covered by Insight Health, 15% were added to the patient numbers derived from ART prescription data. The determined patient numbers were contrasted with numbers of patients under ART in ClinSurv HIV for each of the years 2006 to 2009. The resulting ratio ( = coefficient) was assessed for its robustness over time. The average value was applied to the years 2002 to 2005, for which ART prescription data were unavailable. We assumed that this coefficient, determined for the subpopulation under ART, would also approximate the share of ART naïve individuals enrolled in ClinSurv HIV of all ART naïve HIV-diagnosed individuals living in Germany.

Step 3 was to determine the HIV-prevalence in TB patients. For each of the years 2002 to 2009, HIV/TB case numbers and their confidence limits (step 1) were multiplied with the coefficient determined in step 2 to project the number of HIV/TB cases for entire Germany. These products were divided by the number of notified culture-positive TB patients of the respective years.

To assess the 2002 to 2009 trend in the estimated HIV-prevalence in TB patients, negative binomial regression was used and incidence rate ratios (IRR) including 95%CIs and p-values (5% significance level) were specified.

### Analysis of HIV/TB Patient Characteristics

The HIV/TB patients enrolled in ClinSurv HIV were described as to age, sex, and their geographical region of origin. For continuous variables median and inter-quartile range (IQR), for categorical variables numbers and proportions were specified.

Following a case-cohort approach, characteristics of HIV/TB patients were contrasted with those of the entire ClinSurv HIV cohort enrolled until the end of 2009, as well as with all culture-positive TB patients notified from 2002 to 2009 (pooled).

All analyses were performed using Excel (version 11, Microsoft Corporation, Redmond, Washington, USA) and Stata (version 12.1, StataCorp LP, Texas, USA).

### Ethics Statement

The ClinSurv HIV study has been approved by the Federal Commissioner for Data Protection and Freedom of Information in Germany. The need for written consent was waived given the anonymous reporting of data to the RKI, the Federal Public Health Institute of Germany. German TB notification data are collected within the legal framework of the Protection against Infection Act [21].

## Results

From 1999 to 2009, a total of 15,531 HIV-positive individuals were enrolled in the ClinSurv HIV cohort (recorded as of June 30, 2010). The proportion of patients under ART was 81% to 87% from 2002 to 2009, respectively.

Patient numbers derived from ART prescription data were in average by factor 4.8 higher than numbers of ClinSurv HIV participants under ART. Accordingly, the ClinSurv HIV cohort was assessed to enrol 21% of all HIV-diagnosed individuals living in Germany.

The cohort comprised 298 HIV-positive individuals diagnosed with culture-positive TB from 2002 to 2009 and thus classified as HIV/TB patients. Annual HIV/TB case numbers ranged from 25 (95%CI 16–37) in 2009 to 56 (95%CI 42–73) cases in 2005. Extrapolated to Germany overall this corresponds to 126 (95%CI 81–185) HIV/TB cases in 2009 and 270 (95%CI 204–350) cases in 2005 (Table 1).

**Table 1:** Data underlying the estimation of the HIV-prevalence in culture-positive TB patients in Germany, 2002 to 2009§.

	2002	2003	2004	2005	2006	2007	2008	2009	Overall
**no. cult+ TB** **notifications**	4,965	4,714	4,372	4,116	3,773	3,475	3,163	3,150	31,728
**no. patients in** **ClinSurv HIV**	7,333	7,766	8,277	8,657	8,902	9,264	9,596	9,840	15,531
**no. patients in ClinSurv** **HIV under ART**	5,920	6,343	6,806	7,097	7,359	7,776	8,155	8,523	n.a.
**Proportion of patients in** **ClinSurv under ART (%)**	81	82	82	82	83	84	85	87	n.a.
**no. ART prescriptions**	n.a.	n.a.	n.a.	n.a.	11,553,109	11,254,315	11,802,210	12,352,169	n.a.
**no. patients from ART prescriptions**	n.a.	n.a.	n.a.	n.a.	35,185	36,437	38,730	42,438	n.a.
**Coefficient**	4.80*	4.80*	4.80*	4.80*	4.78	4.69	4.75	4.98	4.80
**no. HIV/TB cases** **[95%CI]**	44 [32–59]	42 [30–57]	41 [29–56]	56 [42–73]	32 [22–45]	26 [17]–[38]	32 [22–45]	25 [16]–[37]	298 [n.a.]
**no. HIV/TB cases** **(extrapolated)**	212[154–284]	202 [146–273]	198 [142–268]	270 [204–350]	153 [105–216]	122 [80–179]	153 [105–216]	126 [81–185]	1,430 [n.a.]
**estimated** **HIV-prevalence in** **cult+ TB (% [95%CI])**	4.3 [3.1–5.7]	4.3 [3.1–5.8]	4.5 [3.2–6.1]	6.6 [5.0–8.5]	4.1 [2.8–5.7]	3.5 [2.3–5.1]	4.8 [3.3–6.8]	4.0 [2.6–5.9]	4.5 [n.a.]

§ data sources include national tuberculosis notification data, the ClinSurv HIV cohort, and ART prescription data by Insight Health; *average of the 2006 to 2009 coefficients; no. = number; n.d. = no data; n.a. = not applicable.

The estimated HIV-prevalence in notified culture-positive TB patients was on average 4.5% in the study period. The annual level ranged from 3.5% (95%CI 2.3–5.1%) in 2007 to 6.6% (95%CI 5.0–8.5%) in 2005, and was 4.0% (95%CI 2.6–5.9%) in 2009 (Table 1).

**Figure 2:**
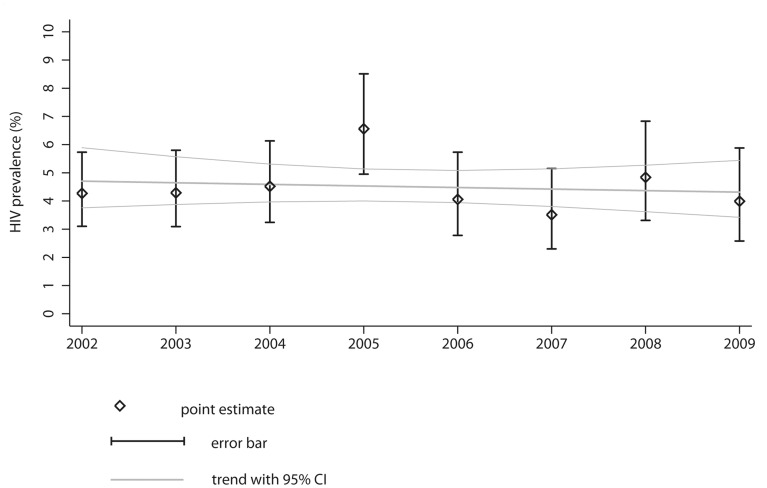
Estimated HIV-prevalence in TB patients in Germany, 2002 to 2009. Point estimates are presented with 95% binomial exact confidence intervals (CI). The estimated trend of the HIV-prevalence in culture-positive TB patients with 95%CI was determined by negative binomial regression. Underlying data sources include national TB notification data, ART prescription data by Insight Health and the ClinSurv HIV cohort.

Negative binomial regression revealed that the estimated HIV-prevalence in TB patients was overall stable over time (IRR 0.99; 95%CI 0.93–1.04; p = 0.659; Figure 2); year-wise regression analysis revealed that the 2005 estimate was an upwards outlier (IRR 1.54; 95%CI 1.28–1.84; p<0.001) using year 2002 as reference.

The 298 HIV/TB patients identified in the ClinSurv HIV cohort were characterized by a male-to-female ratio of 2.1. Their median age was 36 (IQR 30–44) years at enrolment, and 38 (IQR 31–45) years at TB diagnosis; female patients were about eight years younger than males. The HIV/TB patients’ geographic origin was in 41% (117/286 with available information) German and in 59% (169/286) foreign. A foreign origin was markedly more frequent in women than in men (88% (80/91) versus 46% (89/195), respectively; Table 2).

**Table 2:** Case characteristics stratified by sex of HIV/TB patients, of the entire Clinsurv HIV cohort, and among notified culture-positive TB patients in Germany, 2002 to 2009 (pooled data).

Patient characteristics	By sex	ClinSurv HIV cohort		TB notification data
		HIV/TB cases	All HIV cases	All culture-positive TB cases
**Sex ratio (male-to-female)**		2.1	4.0	1.6
**Age at enrolment (m[IQR]** **years; n)**	All	36 [30]–[44]; n = 298	38 [32–45]; n = 15,496	n.a.
	Female	31 [27]–[37]; n = 96	34 [28]–[40]; n = 3,123	n.a.
	Male	39 [32–46]; n = 202	39 [33–46]; n = 12,371	n.a.
**Age at TB diagnosis (m[IQR]** **years; n)**	All	38 [31–45]; n = 298	n.a.	48 [32–68]; n = 31,721
	Female	33 [28]–[39]; n = 96	n.a.	48 [35–66]; n = 12,326
	Male	41 [33–48]; n = 202	n.a.	46 [30–70]; n = 19,367
**Proportion of foreign origin** **(n/N (%))**	All	169/286 (59)	3,659/15,240 (24)	13,793/30,715 (45)
	Female	80/91 (88)	1,476/3,033 (49)	5,796/11,942 (49)
	Male	89/195 (46)	2,183/12,207 (18)	7,976/18,745 (43)

n.a. = not applicable; m = median; IQR = inter-quartile range.

HIV/TB patient characteristics differed from those of the entire ClinSurv HIV cohort, and from those of notified culture-positive TB patients: The entire ClinSurv HIV cohort was characterized by a male-to-female ratio of 4.0, a median age at enrolment of 38 (IQR 32–45) years, and by 24% (3,659/15,240) individuals of foreign origin. Culture-positive TB patients had a male-to-female ratio of 1.6, a median age of 48 (IQR 32–68) years at TB diagnosis, and were in 45% born abroad (Table 2).

**Figure 3:**
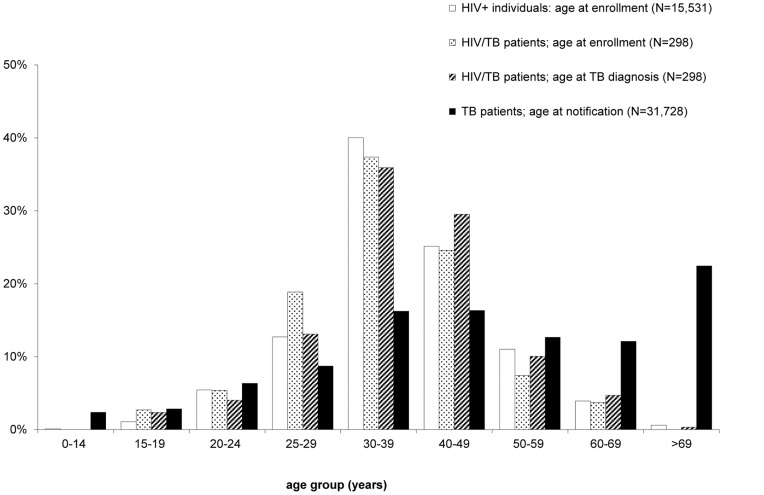
Age distribution among individuals with HIV, TB, and HIV/TB. The HIV and HIV/TB groups refer to individuals enrolled in ClinSurv HIV since 1999; and HIV/TB patients diagnosed with TB from 2002 to 2009. The TB group refers to notified culture-positive TB patients in Germany, 2002 to 2009 (pooled data).

The age distribution of HIV/TB patients, both at enrolment and TB diagnosis, contrasted with the age distributions of the entire ClinSurv HIV cohort at enrolment and of the notified culture-positive TB patients is shown in Figure 3.

**Figure 4:**
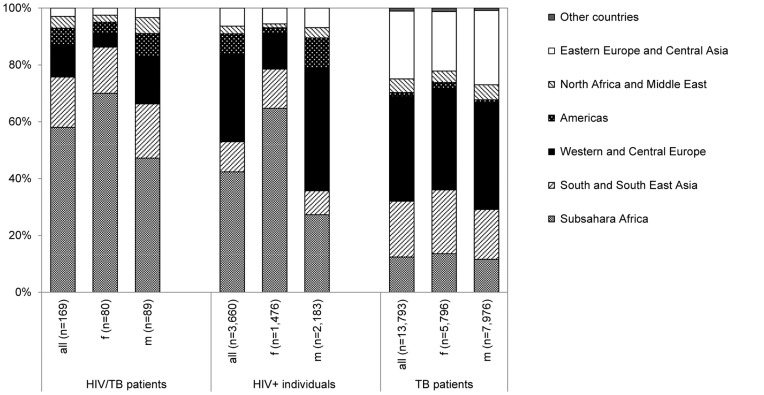
Distribution of foreign origins among individuals with HIV, TB, and HIV/TB stratified by sex. The HIV and HIV/TB groups refer to individuals enrolled in ClinSurv HIV since 1999, and HIV/TB patients diagnosed with TB from 2002 to 2009 with specified foreign origin in terms of predominant stay. The TB group refers to notified culture-positive TB patients in Germany with specified foreign country of birth, 2002 to 2009 (pooled data).

The proportional distribution of regions of foreign origin differed by sex and across HIV/TB patients, the entire ClinSurv HIV cohort, and culture-positive TB patients as shown in Figure 4. PLWH including HIV/TB patients of foreign origin, especially women, were most frequently from Subsahara Africa. Foreign-born TB patients in contrast originated more often from the Western and Central Europe, as well as the Eastern Europe and Central Asia regions; without marked differences between male and female patients.

## Discussion

With this study, we provide, to our knowledge, the first nationwide estimate of the HIV-prevalence in TB patients for Germany, as well as a demographic description of HIV/TB patients.

Core of our estimation was a clinical HIV surveillance instrument, the ClinSurv HIV cohort. ClinSurv HIV was the only identified large-scale data source systematically enrolling HIV-diagnosed individuals and capturing TB as AIDS-defining disease including its date of diagnosis. The cohort was assessed to enrol 21% of all HIV-diagnosed individuals living in Germany.

The estimated level of HIV in TB patients was largely stable level around 4.5% from 2002 to 2009.

For cross-validation of our estimates we identified two epidemiological studies providing quantitative data on HIV in TB patients in Germany. From 2002 to 2004, a multicentre study had been carried out by the German Central Committee against Tuberculosis. Here, 4.3% (101/2.364) of the voluntarily interviewed TB patients (unknown level of HIV testing) were reportedly HIV-positive [30]. This was in agreement with our nationwide 2002 to 2004 estimates of 4.3% (95%CI 3.1–5.7%), 4.3% (95%CI 3.1–5.8%), and 4.5% (95%CI 3.2–6.1%), respectively. From 2007 to 2010, a smaller retrospective case series of TB patients in Berlin prisons revealed a HIV-prevalence of 3.3% (2/60; all tested for HIV) [unpublished data]. This was lower than our respective 2007 to 2009 estimates of 3.5% (95%CI 2.3–5.1%), 4.8% (95%CI 3.3–6.8%), and 4.0% (95%CI 2.6–5.9%) but within the confidence intervals.

Outside of Germany, 15 EU/EEA member states provided 2009 data on the HIV-prevalence among TB patients with known HIV tests status to the European Centre for Disease Prevention and Control (ECDC). Their HIV-prevalence was 7.3% (1,334/18,181) in average, and 4.6% (43/930) in Belgium, 4.3% (9/207) in Denmark, and 3.8% (6/160) the Czech Republic (all neighbouring countries of Germany) [19]. Our estimate for Germany is within the range of these levels. However, assuming the level of HIV testing was unknown (as it is the case in Germany) and thus all TB notifications being used as the denominator would result in a HIV-prevalence of 3.2% (1,334/42,335) in average for these 15 EU/EEA member states, and of 4.6% (43/944) in Belgium, 2.7% (9/337) in Denmark, and 0.9% (6/695) in the Czech Republic. A low proportion of HIV testing, as found for several EU/EEA member states, influences the ascertainable proportion of HIV among TB patients [31] and leads to an underestimation of the comorbidity when the denominator comprises all TB notifications.

Our estimation approach is subject to several limitations. First, ClinSurv HIV centres are mainly based in urban areas [23]. City States and Federal States with big cities are known to have above-average HIV and TB notification rates in Germany [22]. Moreover, the proportion of HIV-positives among TB patients may be increased in big cities, as known from several EU/EEA member states [32], involving potential geographical bias. Secondly, a possible overrepresentation of individuals with AIDS in the ClinSurv HIV cohort, related to the particular expertise of the cohort’s clinical centres, as well as an underrepresentation of individuals originating from HIV high-prevalence countries were suggested by Bätzing-Feigenbaum and colleagues [23] with unknown effect on the cohort’s representativeness for HIV/TB among PLWH. Thirdly, diagnostic bias cannot entirely be ruled out, e.g., a HIV testing shifted towards TB patients at (supposedly) increased HIV risk as observed in a study in the United Kingdom [33]. Though, universal HIV counselling and testing in TB patients is recommended in Germany [34] and awareness is raised on TB among PLWH [35]. Fourthly, the ClinSurv HIV cohort focusses by definition on culture-positive TB, which may not represent the entire TB burden among PLWH and does not correspond to definition used in national TB notification data [27]. If all ClinSurv HIV centres had departed from their TB case definition and considered also clinically diagnosed TB eligible, this would result in a HIV-prevalence in TB patients of around 3.1% for 2002 to 2009 corresponding to an overestimation of about 47% (ranging from 41% to 54%) by restricting the denominator to culture-positive cases.

To account for the uncertainty of our estimates we provided them with 95% confidence intervals. No separate range of uncertainty was, however, determined when assessing the nationwide coverage of ClinSurv HIV.

Beyond validity, the question arises of how important a level of about 4.5% HIV in TB patients is for individual and public health. In different settings, individual disease burden of HIV/TB was assessed to be particularly high involving dual stigma [36], [37], more complex clinical manifestations [12], [13], and complications resulting from simultaneous antituberculous treatment and ART [10], [13], such as immune reconstitution inflammatory syndrome (IRIS) [38]–[40] and high risk of severe liver toxicity [41]. These aspects entail time- and resource-intensive patient care. From this view point, the determined number of about 126 (95%CI 81–185) HIV/TB patients in 2009 represents a non-negligible burden of comorbidity.

Knowledge of the HIV-prevalence in TB patients is not only important in absolute terms but to evaluate trends and epidemiological patterns. We found no significant decline in the estimated HIV-prevalence in a context of decreasing HIV/TB and TB case numbers, but a presumably increasing HIV-prevalence in Germany from 2002 to 2009 [28].

Our general estimate of HIV in TB patients may serve as starting point for group-specific investigations. Evaluating demographic characteristics of HIV/TB patients indicated a shift towards individuals of foreign origin among HIV/TB patients, which supports earlier findings [32], and might reflect the high HIV/TB burden in the regions of origin, mainly in Subsahara African countries [42].

HIV/TB patients were found to have a male-to-female ratio of 2.1, which approximates the ratio of 2.7 found for 16 WHO European region countries (pooled) in 2005 and reflects that both infectious diseases are more prevalent in the male population in the European region [2]. A systematic review of HIV/TB comorbidity in the EU/EEA identified an increased risk of HIV/TB in male TB patients [32]. This is also supported by our identified male-to-female ratio of 1.6 among TB patients being lower than among HIV/TB patients (ratio 2.1). More pronounced, however, was the overrepresentation of women among HIV/TB patients in relation to all PLWH enrolled in ClinSurv HIV (ratio 4.0). TB as AIDS-defining disease was only slightly more frequent in women according to HIV/AIDS notification data of EU/EEA member states [43]. Sex ratios, however, might be influenced by the patients’ geographic origins and their respective epidemiological patterns suggesting the need for multivariable approaches for correct interpretation.

Our study, estimating the HIV-prevalence in TB patients by joint evaluation of available anonymous HIV and TB surveillance data sources, enhances rather than suspends the need for an integrated HIV/TB notification within the national TB surveillance system in Germany. Only this would allow group-specific analyses and the assessment of for instance treatment outcomes and drug-resistance in both HIV-positive and HIV-negative TB patients. Such quantitative evidence is needed to monitor progress in TB control [44], to further define risk-groups for HIV/TB and target interventions accordingly, as well as to design further operational research [3].

Yet, already today, our developed approach for estimating the HIV-prevalence in TB patients is valuable for epidemiological monitoring of HIV/TB and applicable within the current legal frameworks.
